# Postnatal surgical complications in lower urinary tract obstruction following fetal vesico- amniotic shunting

**DOI:** 10.1186/s12887-025-05457-3

**Published:** 2025-02-24

**Authors:** Anna Meßling, Anna-Maria Ziegler, Jochen Hubertus, Christoph Berg, Matthias Nissen, Andreas Heydweiller, Eva Weber

**Affiliations:** 1 Department of Pediatric Surgery, Ruhr- Universität Bochum, Bochum, Germany; 2 Department of Pediatric Surgery, Universitätsklinikum Bonn, Bonn, Germany; 3 Department of Obstetrics and Gynecology, Division of Prenatal Medicine, Universitätsklinikum Köln, Cologne, Germany

**Keywords:** Lower urinary tract obstruction, Fetal surgery, Vesico-amniotic shunt, Neonatal surgery, Congenital urinary tract abnormalities, Shunt complications

## Abstract

**Purpose:**

Fetal lower urinary tract obstruction (LUTO) is a rare congenital disease associated with high morbidity and mortality due to pulmonary hypoplasia and renal insufficiency. Fetal management includes early vesicoamniotic shunting (VAS), a technique that has evolved in recent years to preserve kidney function. Previous publications have focused on intrauterine shunt complications, such as dislocation and preterm premature rupture of membranes (PPROM). In our study we aimed to assess postnatal shunt related complications that became obvious after birth.

**Materials and Methods:**

We describe our preliminary experience with 25 fetuses who underwent VAS with a Somatex^®^ shunt as well as postnatal shunt removal at two experienced centers for fetal medicine. The intrauterine course, underlying pathology, postnatal outcome and details on shunt explantation and related complications were assessed. The data were retrospectively analysed in relation to the intrauterine course, underlying pathology, further malformations, and perioperative characteristics with a focus on the complication spectrum and type of intervention.

**Results:**

Twenty-five fetuses underwent VAS at a median of 17 weeks. Two newborns were excluded because they died within the first 24 hours of life secondary to fulminant lung hypoplasia. In ten (43%) newborns, shunt removal was performed under local anaesthesia. In 13 (57%) neonates, the shunt was explanted surgically, and five (22%) of these operations were defined as complex.

**Conclusion:**

Intrauterine VAS with a Somatex^®^ shunt is feasible, and in the majority of cases, shunt explantation can be easily performed after birth. However, the umbrellas may cause intraabdominal tissue damage, peritoneal adhesions or skin defects, and early surgical management of VAS associated complications might be necessary.

## Introduction

Fetal lower urinary tract obstruction (LUTO) is a congenital anomaly of the kidney and urinary tract, and is among the most frequent causes of end-stage kidney disease in children. The reported incidence of LUTO is 3.3 per 10,000 births, and LUTO predominantly affects male newborns [1].

The most common underlying cause of LUTO is posterior urethral valves, accounting for 63% of cases [2], followed by urethral atresia or obstructive ureterocele. In addition, more complex pathologies, such as cloacal plate anomalies cause an obstruction of the lower urinary tract. However, non-obstructive causes, such as the Prune belly syndrome or megacystis microcolon syndrome (MMIHS), are also known that lead to fetal megacystis.

LUTO leads to disturbed embryonic development of the nephrons and kidney function with high neonatal mortality and long-term morbidity. Prenatal oligo- and anhydramnions are the result of reduced urinary excretion and can result in pulmonary hypoplasia or positional limb abnormalities [3]. Severe pulmonary hypoplasia is likely to cause, if untreated, high mortality rates. [4] Postnatally congenital obstructive uropathy accounts for up to 60% of all pediatric renal transplants [5]. If LUTO remains untreated, live birth rates are low, at 0-15% [6, 7], and up to half of survivors often require dialysis or transplantation due to renal failure [8].

There is evidence that early prenatal diagnosis of fetal megacystis followed by prenatal intervention appears to have a positive impact on neonatal outcomes in terms of morbidity and mortality.

Intrauterine vesicoamniotic shunting (VAS) is the most commonly used method for preventing fetal megacystis,. The “Percutaneous Vesicoamniotic Shunting in Lower Urinary Tract Obstruction (PLUTO)“ trial in 2013 showed that the VAS in the second trimester decreases neonatal mortality [9, 10]. However, in this prospective trial, long-term survival with normal kidney function was rare [10] and the risk of terminal kidney failure with the necessity of dialysis or transplantation was up to 50% [11].

Recent studies have shown that an earlier VAS before >17 weeks might have a positive effect on postnatal renal function and that the duration of the obstruction is a significant predictor of renal impairment [12, 13].

The standard device for a VAS is the Harrison fetal bladder stent (Cook Medical Inc., Bloomington, IN, USA), which has a relatively high rate of secondary dislocation. The Somatex^®^ intrauterine shunt (Hologic Inc., Malborough, Massachusetts, USA) has a different design to reduce the dislocation rate. In addition to the reduced dislocation rate, the major advantage of the Somatex^®^ shunt is that it can be placed through a 18G needle, which is half the diameter of the Harrison introducer with 13G. This allowed the VAS procedure to be performed as early as the first trimester, from 11 weeks onwards. However, VAS using the Somatex^®^ shunt may also not be without complications.

Since numerous publications have already been released on the prenatal course, fetal surgical complications, and the long-term outcomes of children after VAS [6, 10, 12, 14, 15], we have focused on the postnatal surgical complications after vesicoamniotic shunting with the Somatex ® shunt.

## Materials and Methods

Surviving neonates with a prenatal diagnosis of fetal megacystis who received an intrauterine Somatex^®^ shunt in an 8-year period (2014–2022) in one of two participating university hospitals in Germany (Universitätsklinik Bonn and Marien-Hospital Witten; Ruhr- Universität Bochum)) were included.

Megacystis was defined as a longitudinal bladder diameter >15 mm in the first trimester and >25 mm in the second trimester.

Once the patient was diagnosed, additional examinations were performed to rule out further fetal malformations, and an interdisciplinary parental counselling was provided. If indicated, intrauterine vesicoamnial shuning with the Somatex^®^ shunt was applied.

The Somatex^®^ shunt consists of two self-expanding wire mesh umbrellas at both ends and a nitinol wire mesh shaft with an internal silicone coating. The shunt is 25 mm long with an expanded diameter of 2.6 mm. Shunt insertion was performed percutaneously through an 18G puncture cannula under ultrasound guidance in one of the abovementioned hospitals by a senior physician or senior consultant.

Maternal and neonatal electronic databases and charts were reviewed retrospectively for maternal age, time of initial diagnosis, time of VAS, ongoing pregnancy, additional invasive procedures, birth mode, gestational age at delivery, sex, associated anomalies, newborn age at shunt removal and especially shunt complications.

As shown in Table 1, these shunt complications were classified into subgroups: intraabdominal, subcutaneous, or intravesical dislocations; shunt dysfunctions; iatrogenic abdominal wall defects; intestinal adhesions; and skin defects.

**Table 1:** Epidemiologic characteristics

**Sum of patients** (n (%))	23 (100)
**Diagnosis after birth**	
-PUV	16 (70)
-Urethral atresia	5 (22)
-MMIHS	1 (4)
-Megacystis-megaureter-syndrome	1 (4)
**Re-shunting**	10 (43)
**Shunt extraction**	
Non-surgical	10 (43)
Surgical	13 (57)
Complicated surgery	5 (22)
**Shunt complications**	16 (70)
-Shunt dislocations	15 (65)
-Shunt dysfunctions	3 (13)
-Iatrogenic abdominal wall defects	3 (13)
-Intestinal adhesions	4 (17)
-Bladder adhesions	1 (4)
-Urinary ascites	2 (9)
-Aplasia cutis	1 (4)
**Associated malformations**	6 (26)
Prune belly syndrome	3 (13)
*Anal atresia*	2 (9)
*MMIHS*	1 (4)

*PUV* posterior urethral valves, *MMIHS* megacystis microcolon syndrome.

Due to postnatal findings, we also compared subgroups based on the underlying disease, presence of other malformations or presence of overriding syndromic disorders, which are described in Table 1.

Special attention was given to the mode of postnatal removal of the shunt. Shunt removal was categorized as surgical or nonsurgical. Surgical procedures, in turn, were categorized into complicated and uncomplicated operations. Whereas the uncomplicated procedures included only the surgical shunt removal, we defined complicated shunt removals as those that required an additional shunt-associated procedure such as bowel resection, abdominal fascial reconstruction or bladder repair during surgery for shunt removal. Table 2 summarizes these results.

**Table 2:** Surgical vs. non-surgical shunt removal

**Shunt removal**	**Surgical**	**Non-surgical**	**p**
n (%)	13 (57)	10 (43)	
GA at LUTO diagnosis (weeks)	16.8±1.3	16.8±1.4	0.99
GA at first shunting (weeks)	16.9±4.8	17.2±4.5	0.89
GA (weeks)	36±2.2	37.6±1.9	0.07
Maternal age (years)	31.4±5.1	31±4.8	0.86
Re-shunting (n (%))	9 (69)	1 (10)	0.045
Associated malformations (n)	6 (46)	0	0.019
amnion refilling (n)	8 (62)	5 (50)	0.69

*GA* gestational age, *LUTO* lower urinary tract obstruction.

Patients who underwent VAS before 14+6 weeks of gestation were categorized into the early VAS group, and those receiving a VAS from 15+0 gestational weeks onwards were categorized into the late VAS group.

Data acquisition was performed with Microsoft Excel© (Microsoft Corporation, Redmond, WA, USA) and statistical analysis was performed with OriginPro2021© software (OriginLab, Northampton, MA, USA). A normal distribution was confirmed by Kolmogorov-Smirnov test. Comparisons of paired parametric values, expressed as the means ± standard deviations (SD), were performed by Student’s t- test. Categorical variables are presented as frequencies and percentages and were compared by Fisher’s exact test. A p value ≤ 0.05 was considered to indicate a significant difference.

## Results

We identified 25 fetuses that underwent VAS at a median of 17 weeks with a mean gestational age at diagnosis of 16.7 weeks. Two newborns were excluded because they died within the first 24 hours of life secondary to fulminant lung hypoplasia.

Fetal megacystis was diagnosed up to the end of the 14^th^ week of gestation in 7/23 (30%) patients with subsequent early VAS. Late VAS was performed from the 15^th^ week onwards in 16/23 (70%) patients.

### Epidemiology

The most common underlying disease was posterior urethral valves (PUV) in 16/23 (70%) patients, followed by urethral atresia or stenosis in 5/23 (22%) patients, MMIHS and Megacystis-megaureter syndrome in 1/23 (4%) patient each.

While isolated LUTO occurred in 17/23 (74%) patients, additional malformations (Prune-belly syndrome (*n*= 3; 13%); anal atresia (*n*= 2; 9%) and MMIHS (*n*= 1; 4%)) were found in 6/23 (26%) patients. The epidemiologic characteristics of the study population are summarized in Table 1.

### Re-Shunting and shunt complications

As described in previous publications, complications following VAS were not infrequent in our cohort. Although there were no immediate complications, such as bleeding or infection, we recorded fetal shunt complications in 16/23 (70%) patients.

The most frequent shunt complication was shunt dislocation, which occurred in 15/23 (65%) fetuses and caused intrauterine re-shunting in 10/23 (43%) fetuses.

In the early VAS group, 6/7 (86%) of patients developed shunt complications throughout pregnancy, whereas in the late VAS group, 10/16 (63%) patients developed shunt complications.

The intraabdominal dislocation of the shunt caused intestinal adhesions in 4/23 (17%) patients, 3/23 (13%) of whom were in the early VAS group and 1/23 (4%) of whom was in the late VAS group. Dislocations into the bladder were observed in 1/23 (4%) patient in the early VAS group.

Urinary ascites as a consequence of shunt dislocation and drainage of urine into the abdominal cavity was observed in 2/16 (13%) fetuses of the late VAS group.

Shunt dysfunction occurred in 3/23 (13%) fetuses; 2/7 (29%) in the early VAS group and 1/16 (6%) in the late VAS group.

In our series, we detected 3/23 (12%) fetal iatrogenic abdominal wall defects, all with omental herniation (1/7 (14%) in the early VAS group and 2/16 (13%) in the late VAS group).

Chronic skin friction by the umbrellas of the Somatex^®^ shunt induced a skin defect similar to an aplasia cutis in 1/23 (4%) case from the late VAS group, located at the upper extremity.

### Preterm labour and maternal complications

A well-known maternal complication of VAS is preterm rupture of membranes (PROM), which occurred in 2/23 (8%) of pregnancies, with one case in each subgroup. In 1/23 (4%) case, the shunt was incorporated into the uterus wall and had to be removed endoscopically.

Preterm labour occurred in 7/23 (30%) live births. The mean gestational age (GA) in the early VAS group was 37.1±1.2 weeks, and that in the late VAS group was 36.5±2.5 weeks.

### Surgical and non-surgical shunt removal

Our particular focus was on differentiating between surgical and non-surgical shunt removal. Within the cohort, 10/23 (44%) shunts could be removed under local anaesthesia. In 13/23 (56%) newborns, the shunt was explanted surgically, and 5/23 (21%) of these surgeries were complex. Subgroup analysis revealed that within the early VAS group, 4/7 (57%) neonates required surgical shunt explantation, with 2/4 (50%) surgeries resulting in complications. Postnatally, 9/16 (56%) patients with late VAS needed surgical shunt extraction. These removals were found to be complicative in 3/16 (19%) patients .

The second significant factor was the high rate of re-shunting among the shunts that would later require surgical explantation. Eight out of 13 were re-shunted patients. Only 2/10 (20%) patients in the non-surgical subgroup underwent intrauterine re- shunting (see Table 2).

### Additional fetal malformations

We investigated a cohort of individuals with associated malformations and found that 6/23 (26%) neonates had additional malformations. All 6 patients required a surgical shunt removal, of which 3/6 (50%) were explanted via laparotomy, of which 1/3 (33%) was classified complicated. Malformations were observed predominantly in the late VAS group.

### Early vs. late VAS

Detailed analysis revealed that 4/7 (57%) patients in the early VAS group underwent surgical removal of the Somatex^®^ shunt, with 2/4 (50%) complicated procedures. Within the late VAS shunt placement group, 9/16 (56%) neonates required surgical removal, of which 3/9 (33%) cases were complicated (Table 1)."

The age at 1^st^ intervention was 2.6 days for the entire cohort; for the early VAS group, it was 3.1 days, and for the late group, it was 2.3 days.

## Discussion

Postnatal surgical management after intrauterine VAS in fetuses with megacystis is not standardized and has rarely been reported. Here, we reported our experience in the management of postnatal complications directly associated with VAS removal.

In 1984, Glick et al. [16] and in 2003, a research group led by Kitagawa [17] demonstrated in animal studies that the severity of renal damage depends on the timing of onset of obstruction during pregnancy, as well as the duration and extent of bladder obstruction, suggesting that early resolution of the obstruction can lead to reversible kidney damage resulting in normal renal function.

Since it has additionally been recognized that the initial signs of renal dysplasia can be detected from the 15^th^ week of pregnancy onwards, studies have been conducted to analyse earlier VAS placement before 14 weeks of gestation.

With the introduction of the Somatex^®^ shunt, earlier shunt placement was feasible due to the smaller size of the introducer. Furthermore, this shunt exhibited significantly lower dislocation rates due to the presence of parasols on both ends.

In 2020, Strizek et al. demonstrated that early VAS before the 14th week reduces pulmonary hypoplasia and is also nephroprotective with a comparable complication profile to that of later shunt insertions. The possibility of early shunting in these small fetuses was also demonstrated by Strizek et al. [18], who provided a comparison of the two most common stents in Germany. Moreover, the Somatex^®^ intraunterine shunt showed significantly lower dislocation rates in the early VAS than did the Harrison fetal bladder stent (36.4% vs. 87.5%). Overall, this resulted in a high neonatal survival rate of 81.8% in the Somatex^®^ group.

In our cohort of initially 25 fetuses, two neonates died on the first day of life due to fulminant pulmonary hypoplasia and were excluded from further analysis. The surviving 23 children were treated with the Somatex^®^ shunt. Since numerous articles have already been published that analysed the various shunting procedures, we focussed on those cases with postnatal surgical complications secondary to VAS and further characterized those patients actually requiring postnatal surgical intervention. While ten shunts could be removed under analgosedation, 13 shunts had to be removed surgically. Among these, five were removed laparoscopically and eight were removed via laparotomy.

We were aware of the possible shunt-related complications, which are reported to occur in up to 45% of patients [11]. Our data analysis revealed that 70% of all shunt placements are likely to cause complications due to vesicoamniotic shunting. In particular, the rate of shunt complications, reaching 86%, was high in the early VAS group, whereas the complication rate of intrauterine shunting in the late VAS group was approximately 60%. However, all 7 early shunted children survived the perinatal period.

In most of our patients, these complications were shunt dislocations, accounting for 65% of all complications. Strizek et al. [15] reported a 40% shunt dislocation rate in a smaller cohort of 10 patients, whereas Stadié et al. [19] also described shunt complications in 81% of their patients. As previously known, other shunt-related complications might include shunt blockage in 25% of patients and shunt migration in 20% of patients, as well as preterm labour and iatrogenic gastroschisis [11].

According to our retrospective analysis, the observed dislocations were commonly intra-abdominal dislocations, resulting in intestinal adhesions in 4 patients. These intestinal adhesions required an adhesiolysis in two neonates and one child needed suturing of the intestine. Adhesion to the bladder wall occurred in one patientwith intravesical shunt dislocation and the removal required a bladder suture. The risk of fetal urinous ascites, followed by subsequent pulmonary impairment, was detected in two patients. In two cases, a partial omentum resection was necessary due to shunt dislocation. The shunt dislocations resulted in operative shunt removal in nine children (60%), including six laparotomies and three laparoscopic shunt extractions.

Our focus turned to the question of which neonates had complicated surgical shunt removals, meaning not only the actual removal of the Somatex^®^ shunt by itself, but also requiring further surgical measures such as adhesiolysis or intestinal suturing.

We identified three subgroups that exhibited notably frequent shunt complications and subsequently more often required surgical shunt removal: the early VAS group, the re- shunted patients and those with additional malformations.

In our cohort, seven fetuses received VAS prior to completing the 15^th^ week of gestation for fetal megacystis. In this early VAS group, 6/7 (86%) children had shunt complications, in the majority shunt dislocations combined with other complications such as shunt dysfunction (n= 2) or iatrogenic abdominal wall defects (n= 1). The complication rate of intrauterine shunting in the late VAS group was approximately 60%. However, all seven early shunted children survived the perinatal period.

Meanwhile, fetuses are being shunted earlier, so the shunts, having a longer duration of placement, are prone to more complications. Therefore, it is understandable that in our cohort 86% of the early VAS group recorded shunt complications. It is emphasized that earlier shunting appears to be associated with complications, but it does not seem to affect perinatal survival*.* As Table 3 indicates, maternal age or gestational age did not significantly differ between the early VAS and late VAS groups.

**Table 3:** Early vs. late VAS

**VAS**	**Early**	**Late**	**p**
n (%)	7 (30)	16 (70)	
GA (weeks)	37.1±1.2	36.5±2.5	0.41
GA at LUTO diagnosis (weeks)	13.3±0.5	18.3 ±4.7	<0.001
Maternal age (years)	30.7±3.8	31.4±5.3	0.72
GA at re-shunting (weeks)	25±7.8 (n= 3)	24±5.9 (n= 7)	0.83

*VAS* vesicoamniotic shunting, *GA* gestational age, *LUTO* lower urinary tract obstruction.

Also Stadie et al. confirmed a generally increased occurrence of shunt complications with early shunt placement, especially before the 17^th^ week of gestation (17.3 vs. 19.8 weeks). The described complications in their cohort are not necessarily associated with non-survival or an increased risk of renal damage [19].

Theory of increased shunt complications in early VAS patients is in line with with observations made by Ruano et al. that severe LUTO is associated with earlier manifestations and that a longer duration of shunt placement can lead to increased shunt-related complications, often necessitating postnatal surgical interventions, reflecting our results.

With complications, especially dislocations, re-shunting of the bladder might become necessary. Thus, 10/23 (43%) fetuses required an additional intrauterine shunt.

Therefore, in the group with operative shunt extractions, in addition to the correlation with early shunt placement before the 15^th^ week of gestation, an increase in the number of re-shuntings in this group of patients of 69% was detected.

In our cohort, ten fetuses required more than one shunting procedure during pregnancy. Of these, nine shunts (90%) had to be surgically removed postnatally, five (50%) of which underwent laparotomy and five (50%) of which involved intraoperatively complicated shunt extractions.

Therefore, from our observations, it can be inferred that ta predictive factor for complicated postnatal VAS removal might be the need for more than one intrauterine VAS procedure. Strizek et al. reported an almost equally high rate of re-shunting of 33% [12]. A 19.5% rate of re-shunting due to shunt dislocation with subsequent need for a second or third intrauterine shunt was also described by Gottschalk et al. [20].

Lissauer et al. from Birmingham [21] noted the potential consequences that repetitive shunt insertions may have after shunt dislocation, such as an increased risk of infection and possible bladder leakage with subsequent urinary ascites. The increased rate of surgical explant shunts in children with multiple shunt placements during pregnancy can be explained by dislocations or adhesions and, as a result, increased complications in fetuses that required more than one intrauterine shunt.

The perinatal outcome of LUTO patients is directly related to the presence of additional malformations, and it has been demonstrated that the severity of additional malformations determines perinatal mortality and morbidity [22]. We were able to define the presence of additional malformations or underlying syndromes as another predictive factor for surgical shunt removal.

The data analysis revealed that all six neonates (100%) with additional malformations needed surgical shunt extraction (*n*= 3 via laparotomy). Further details on additional malformations have already been mentioned in the results section and are shown in Table 1.

Prune belly syndrome and megacystis microcolon hypoperistaltic syndrome (MMHS) can also show megacystis in preterm ultrasounds, but without obstruction of the lower urinary tract [23], and of course, these patients do not profit from fetal shunting; once again, the correct assessment of megacystis during the prenatal period is crucial.

Considering the surviving neonates in our cohort, urethral atresia was the underlying pathology for bladder outlet obstruction in four out of six patients in the group with further malformations, as PUV and MMIHS were reported in one patient each.

The surgical shunt extractions of these children with underlying syndromic conditions or other malformations, however, were not classified as complicated in any of the six cases, even though additional procedures, such as a colostomy, were necessary intraoperatively. This can be attributed not to a complicated VAS extraction but to the ARM. According to the literature, while fetuses with additional malformations often present with severe LUTO, all shunts had to be surgically removed in our cohort; however, removal of the shunts was feasible, and the perioperative period was uneventful.

Therefore, the high number of shunt-related surgical complications does not preclude the use of this technique in fetuses with LUTO.

The highlighted predictive factors of a potentially complicated course after VAS could be utilized to identify patients requiring special attention in postnatal management. These patients and their families should be prenatally connected to a specialized centre capable of providing appropriate care for these little patients since these patients face persistent challenges, necessitating careful consideration and meticulous surgical intervention.

In conclusion, we assume that due to the lack of alternative early therapeutic options and the promising studies that presented favorable outcomes with early VAS placement, one should not refrain from early VAS placement based solely on surgical shunt removal. Furthermore, the observed complications can be managed well during the initial intervention.

## Data Availability

Data is provided within the manuscript or supplementary information files.

## References

[1] Malin G, Tonks AM, Morris RK, Gardosi J, Kilby MD. Congenital lower urinary tract obstruction: a population- based epidemiological study. BJOG. 2012;119:1455–64. 10.1111/j.1471-0528.2012.03476.x. 22925164 10.1111/j.1471-0528.2012.03476.x

[2] Schierbaum, Luca M et al. “Genome-Wide Survey for Microdeletions or -Duplications in 155 Patients with Lower Urinary Tract Obstructions (LUTO).” Genes (2021) 10.3390/genes12091449 10.3390/genes12091449 PMC8468665 34573432

[3] Merrill DC, Weiner CP. Urinary tract obstruction. In: Fisk NM, Moise KJ, editors. Fetal therapy: invasive and transplacental. Cambridge: Cambridge University Press; 1997. p. 273–86.

[4] Freedman AL, Johnson MP, Gonzalez R. Fetal therapy for obstructive uropathy: past, present.future? Pediatr Nephrol. 2000;14(2):167–76. 10.1007/s004670050035. 10.1007/s004670050035 10684370

[5] Morris RK, KS Khan, Kilby MD. Vesicoamniotic shunting for fetal lower urinary tract obstruction: an overview. Arch Dis Child Fetal Neonatal ED 2007; 92: F166-168. 10.1136/adc.2006.099820 10.1136/adc.2006.099820 PMC2675321 17449853

[6] Osborne, Newton G et al. Fetal megacystis: differential diagnosis. Journal of ultrasound in medicine : official journal of the American Institute of Ultrasound in Medicine. 2011;30(6):833-41. 10.7863/jum.2011.30.6.833. 10.7863/jum.2011.30.6.833 21632999

[7] Fievet L, Faure A, Coze S, Harper L, Panait N, Braunstein D et al. Fetal megacystis: etiologies, management, and outcome according to the trimester. Urology. 2014:185-90. 10.1016/j.urology.2014.02.018. 10.1016/j.urology.2014.02.018 24745800

[8] Stadié R, Strizek B, Gottschalk I, Geipel A, Gembruch U, Berg C. Intrauterine vesicoamniotic shunting for fetal megacystis. Arch Gynecol Obstet. 2016:1175-82. 10.1007/s00404-016-4152-4. 10.1007/s00404-016-4152-4 27394921

[9] Ruano R, et al. Fetal lower urinary tract obstruction: proposal for standardized multidisciplinary prenatal management based on disease severity. Ultrasound Obstet Gynecol. 2016;48:476–82. 26690832 10.1002/uog.15844

[10] Morris RK, Malin GL, Quinlan-Jones E, Middleton LJ, Hemming K, Burke D, et al.; Percutaneous vesicoamniotic shunting in Lower Urinary Tract Obstruction (PLUTO) Collaborative Group. Percutaneous vesicoamniotic shunting versus conservative management for fetal lower urinary tract obstruction (PLUTO): a randomised trial. Lancet. 2013; 382(9903): 1496–50 10.1016/S0140-6736(13)60992-7 PMC3898962 23953766

[11] Strizek B, Gottschalk I, Recker F, Weber E, Flöck A, Gembruch et al. Vesicoamniotic shunting for fetal megacystis in the first trimester with a Somatex intrauterine shunt. Arch Gynecol Obstet. 2020;302:133–40. 10.1007/s00404-020-05598-z PMC7266802 32449061

[12] Strizek B, Spicher T, Gottschalk I, Böckenhoff P, Simonini C, Berg C, et al. Vesicoamniotic Shunting before 17 + 0 Weeks in Fetuses with Lower Urinary Tract Obstruction (LUTO): Comparison of Somatex vs. Harrison Shunt Systems. J Clin Med. 2022;11:2359. 10.3390/jcm11092359 10.3390/jcm11092359 PMC9101314 35566484

[13] Bildau J, Enzensberger C, Degenhardt J, Kawecki A, Tenzer A, Kohl et al. Lower Urinary Tract Obstruction (LUTO) – Krankheitsbild, pränatale Diagnostik und Therapiemöglichkeiten. Zeitschrift für Geburtshilfe und Neonatologie. 2014;218(1): 18–26. 10.1055/s-0034-1367042. 10.1055/s-0034-1367042 24595911

[14] Keil C, Bedei I, Sommer L, Koemhoff M, Axt-Fliedner R, Köhler S, Weber S. Fetal therapy of LUTO (lower urinary tract obstruction) - a follow-up observational study. J Matern Fetal Neonatal Med. 2022;35(25):8536–43. 10.1080/14767058.2021.1988562. 10.1080/14767058.2021.1988562 34652254

[15] Fetal therapy of LUTO (lower urinary tract obstruction) – a follow-up observational study. J Matern Fetal Neonatal Med. 2022;35(25):8536-43. 10.1080/14767058.2021.1988562. 10.1080/14767058.2021.1988562 34652254

[16] Kohaut J, Fischer-Mertens J, Cernaianu G, Schulten D, Holtkamp G, Kohl S et al. Postnatal surgical treatment and complications following intrauterine vesicoamniotic shunting with the SOMATEX® intrauterine shunt. A single center experience. J Pediatr Urol. (2023):S1477-5131(23)00277-2. 10.1016/j.jpurol.2023.06.027. 10.1016/j.jpurol.2023.06.027 37451915

[17] Glick PL, Harrison MR, Adzick NS, Noall RA, Villa RL. Correction of congenital hydronephrosis in utero IV: in utero decompression prevents renal dysplasia. J Pediatr Surg. 1984;19(6):649–57. 10.1016/s0022-3468(84)80348-6. 6520669 10.1016/s0022-3468(84)80348-6

[18] Piscione TD, Rosenblum NG. The moelcular control of renal branching morphogenesis: current knowledge and emerging insights. Differentiation. 2002;70:227–46. 12190985 10.1046/j.1432-0436.2002.700602.x

[19] Bierkens AF, Feitz WF, Nijhuis JG, de Wildt MJ, Flos MS, de Vries JD. Early urethral obstruction sequence: a lethal entity? Fetal Diagn Ther. 1996;11(2):137–45. 10.1159/000264293. 8838771 10.1159/000264293

[20] Smith-Harrison LI, Hougen HY, Timberlake MD, Corbett ST. Current applications of in utero intervention for lower urinary tract obstruction. J Pediatr Urol. 2015:341-7. 10.1016/j.jpurol.2015.07.012. 10.1016/j.jpurol.2015.07.012 26441047

[21] Freedman, A L et al. “Long-term outcome in children after antenatal intervention for obstructive uropathies.” Lancet. 1999: 374-7. 10.1016/S0140-6736(98)11006-1 10.1016/S0140-6736(98)11006-1 10437866

[22] Lissauer D, Morris RK, Kilby MD. Fetal lower urinary tract obstruction. Semin Fetal Neonatal Med. 2007;12(6):464–70. 10.1016/j.siny.2007.06.005. 17761463 10.1016/j.siny.2007.06.005

[23] Pringle KC, Zuccollo J, Kitagawa H, Koike J, Delahunt B. Renal dysplasia produced by obstructive uropathy in the fetal lamb. Pathology. 2003;35(6):518–21. 10.1080/00313020310001619145. 14660103 10.1080/00313020310001619145

